# Equivalence of Stock-Recruitment Functions and Parent-Progeny Relationships in Discrete-Time Multi-Stage Models

**DOI:** 10.1007/s10441-025-09493-5

**Published:** 2025-03-14

**Authors:** Ute Schaarschmidt, Anna S. J. Frank, Sam Subbey

**Affiliations:** 1 Department of ICT and Natural Sciences, Norwegian University of Science and Technology (NTNU), PB 1517, NO-6025 Ålesund, Norway; 2 Computational Biology Unit (CBU), Dept. of Informatics, University of Bergen (UiB), PB 7803, NO-5020 Bergen, Norway; 3 Institute of Marine Research, PB 1870, NO-5817 Bergen, Norway

**Keywords:** Population dynamics, Stage-structured models, Recruitment, Fish life cycle, Logic

## Abstract

Understanding the relationship between adult fish populations (the "stock") and the number of new fish entering the population (the "recruits") is essential for effective fisheries management. Traditionally, this relationship is represented by a stock-recruitment (SR) function, which is a simplified mathematical model that directly links stock size to recruitment. However, fish populations pass through several life stages, each stage influenced by unique population dynamic factors. Current SR functions often overlook these complexities, assuming that recruitment depends solely on the adult population size. In this study, we use a multi-stage, age-structured discrete-time population dynamic model that accounts for all life stages and the transitions between them. We demonstrate that, in general, a closed-form, univariate SR function may not accurately represent the recruitment process when these life stages are considered. Instead, we identify specific mathematical conditions under which a SR function is equivalent to our multi-stage model. Our findings suggest a re-evaluation of conventional SR models, advocating for multi-stage approaches to support fisheries management decisions.

## Introduction

**Fig. 1:**
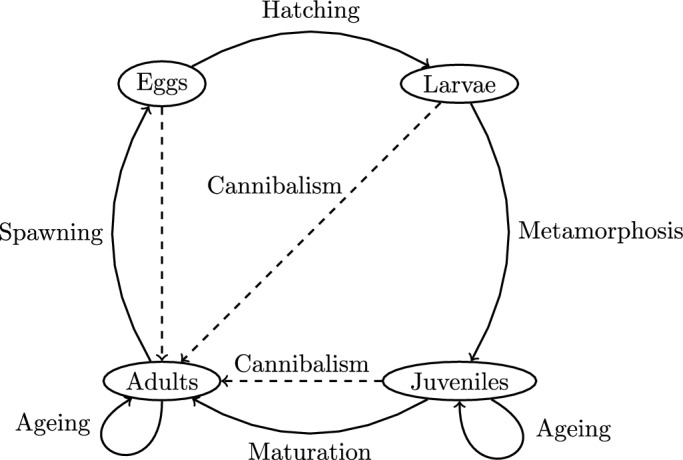
A schematic diagram of the life-history cycle of a fish with four stanzas. The principal developmental processes are indicated. Transitions from one age class to the next age class are represented by loops. In addition, the linkages between the adults and the early life-history stages through cannibalism are illustrated

The life history of marine populations often comprises a series of distinct stages or stanzas (Paulik 1973), each characterized by a unique set of factors influencing survival (Nash 1998) (see Fig. 1).

These life stanzas encompass critical developmental phases such as eggs, larvae, juveniles, and adults. Principal developmental processes (e.g., spawning, hatching, metamorphosis, and maturation) cause transitions from one life stanza to the next (Paulik 1973).

Understanding this intricate dynamics of marine populations is fundamental for effective and sustainable fisheries management (to maintain healthy fish populations). Central to this understanding is the concept of stock recruitment (SR) and the SR function.

Fish stock recruitment refers to the relationship between the number of juvenile fish (recruits) that enter a population and the number of adult fish (the stock) that spawn to produce those recruits. It is a critical concept in fisheries biology and management because it helps (scientists and managers) understand and predict how changes in the number of adult fish (e.g., due to fishing or environmental factors) can affect the abundance of young fish that join the population. The stock recruitment relationship is often described by a function of one variable and defined in terms of numbers of individuals (Chambers and Trippel 1997). In fisheries science, the functions are usually unimodal and often assume that as the size of the parental population increases, the number of recruits also increases or remains constant (Beverton and Holt 1957) and may only decrease after reaching a unique maximum (Ricker 1954). These functions can capture certain patterns and can be useful simplifications. While neither adult population numbers nor recruitment can be measured directly in marine systems, collecting information on prerecruits is even more challenging. SR functions can be fitted to data for mature fish, thereby omitting any challenges related to collecting information on fecundity, egg survival, larvae survival, and juvenile survival from marine systems. This reliance on adult population numbers, not directly measured from the ecosystem, partly explains the popularity of traditional SR functions. However, issues have been raised, which revolve around both the fundamental existence and formulation of these functions (Subbey et al 2014).

The SR function is conventionally assumed to exist. However, several studies have illuminated nuances in recruitment patterns, some of which are inconsistent with conventional SR functions (reviewed by Haddon (2011) and Hilborn and Walters (1992, chapter 7)). For instance, empirical multi-modal patterns of recruitment have been reported in the literature (Hennemuth et al 1980), which may only be explained by multi-modal fish SR functions. Such functions may be derived by considering multiple life stages prior to recruitment  (Brooks and Powers 2007; Paulik 1973), though this is a departure from current convention. On the other hand, multi-stage model simulations have revealed that the existence of a SR function may not be guaranteed under certain conditions. For instance, when parameters such as fecundity, reproductive rates, or predation rates vary with age, the traditional SR function may fail to emerge (Touzeau and Gouzé 1998). In contrast, in multi-stage models that account for recruitment as a function of an age-structured parental population, the resulting function becomes multivariate, diverging from the conventional SR function, which typically relies on a single variable (Schaarschmidt et al 2018). Thus, the quest of the paper is to determine necessary and sufficient conditions for existence of a (I) closed-form SR function, and (II) multivariate SR function in a multi-stage framework.

We do not aim to investigate whether a causal relationship exists between parent and progeny; rather, we assume that such a biological link is present. Our focus is to examine the conditions under which this parent-progeny relationship can be adequately represented by a traditional closed-form SR function, such as the Ricker model (Ricker 1954) or the Beverton-Holt model (Beverton and Holt 1957). We consider recruitment as a function of spawning stock biomass to be a simplified representation of the parent-progeny relationship compared to more complex stage- and age-structured population dynamics models.

In addressing the above issues, the paper uses theorems, which are summarized in a Ph.D thesis (Schaarschmidt 2018). This paper extends the work in Schaarschmidt (2018) by providing rigorous proofs and biological interpretations of the theorems. Furthermore, we discuss the choice of the mathematical model and interpretations of our results.

We will employ a systematic, modeling-driven approach to comprehend the parent-progeny relationship within multi-stage population dynamics. In contrast to studies that rely on inherently variable and uncertain data to investigate SR relationships (Myers and Barrowman 1996; Gilbert 1997), our methodology consistently provides a mathematical representation of the parent-progeny relationship.

Our approach involving research on multi-stage SR relationships is aligned with literature (Quinn and Deriso 1999; Schaarschmidt et al 2018; Touzeau and Gouzé 1998), but differ in the following way. In contrast to (Schaarschmidt et al 2018; Touzeau and Gouzé 1998), we consider all life history stages (eggs, larvae, juveniles and adults), rather than a broad classification of life stages into prerecruits and recruits. We consider a more comprehensive life cycle process than in Quinn and Deriso (1999).

The article is organized in the following way. Section 2 presents the modeling framework adopted in this manuscript. It states the necessary assumptions and the equations of the general discrete time multi-stage model that is used to simulate the entire life history cycle of fish populations. Section 3 focuses on the parent-progeny relationship and its mathematical simplification into the multivariate and closed-form SR functions, by proving necessary and sufficient conditions for their existence. We also provide mathematical proofs of these conditions. In the discussion, Section  4, we compare our findings to current literature and provide biological interpretations for the necessary and sufficient conditions.

## Modeling framework

An adopted modeling framework must satisfy certain criteria, which include the ability to clearly delineate different life stages, e.g., in Fig. 1. This specificity enables a more accurate representation of the dynamics at, and between each stage. While it is possible to categorize the adult population by length (Callahan et al 2019) or size (weight) (Meng et al 2013), we align with previous research on multi-stage SR relationships (Schaarschmidt et al 2018; Touzeau and Gouzé 1998), and focus on an age-structured adult population model.

We now specify a multi-stage model to describe the life-history cycle illustrated in Fig. 1, which is subject to the following biological (**B1–B2**) assumptions and model-time characteristics (**T1–T4**):

**B1:** Egg production varies with fecundity and the proportion of spawners.
**B2:** Mortality rates of larvae and juveniles are functions of numbers of adults, larvae, and juveniles.

The assumption **B1** follows a standard approach in fisheries (e.g. Hilborn and Walters 1992, chapter 3), while **B2** reflects that survival may be affected by processes such as cannibalism, food availability, and competition (e.g. Hilborn and Walters 1992, chapter 7).

**T1:** Discrete Time Transitions: We assume discrete transitions between life stanzas (see e.g., Fig. 1).
**T2:** Unified Time Steps: We consider uniform time steps across all life stanzas.
**T3:** Time Delay Constraint: Our approach incorporates a positive time delay, recognizing the delay between spawning and recruitment.
**T4:** Transition Time: Spawning and transition to the juvenile stage are assumed to happen within one time step. If surviving, juveniles and adults age by one in every simulation time step.

The assumptions **T1**, **T2** and **T3** are consistent with the fisheries literature (Quinn and Deriso 1999, chapter 5). Assumptions **T1** and **T2** also allow for direct comparison between model predictions and observed data, as empirical data on marine populations often come in discrete time intervals. Assumption **T4** can be justified from the fisheries literature, with egg and larva stage duration of a few months, maturation after a few years, and life spans of several years (Petitgas et al 2013). Furthermore, faster evolution of prerecruits in comparison to adults is an assumption underlying other multi-stage models for SR (Schaarschmidt et al 2018; Touzeau and Gouzé 1998).

### A general discrete time multi-stage model (DTMM)

**Table 1:**
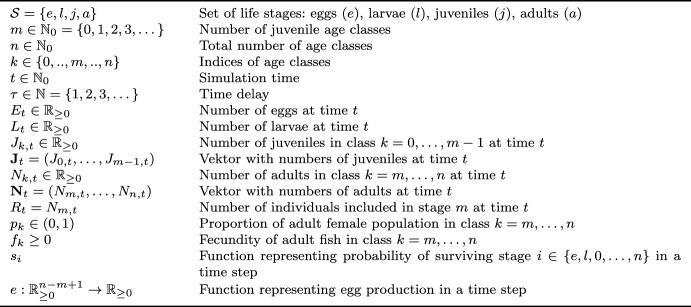
Nomenclature for the DTMM (adapted from Schaarschmidt (2018)).

Based on **B1–B2** and **T1–T4**, we formally define an age-structured population dynamic model that describes births, survivals, and transitions from one stanza to the next. The considered stages are eggs (*e*), larvae (*l*), juveniles (*j*), and adults (*a*). We have age classes $0, \dots , m-1$ representing juveniles and age classes $m, \dots , n$ for adults. We use the symbols in brackets and $0, \dots , n$ as indices. An overview of the nomenclature for the model is given in Table 1.

Let ${\mathcal {D}} \subset {\mathbb {R}}_{\ge 0}^{{n}+1}$, where ${\mathcal {D}}$ is non-empty. The general discrete time multi-stage model (DTMM) is defined by (1)–(7) with initial condition $({\textbf{J}}_0, {\textbf{N}}_0) \in {\mathcal {D}}$.

Most age and / or length structured marine populations have mature individuals distributed over several age and length groups (see e.g., Jokar et al (2021)). Hence, total egg production in (1) is defined as the sum of eggs produced over all age classes and depends on the adult population. The number of larvae presented in (2) is the number of eggs that survive in this time step.

1 $$\begin{aligned} {E}_{t}&= \; \sum \limits _{k= {m}}^{{n}} f_k\;p_k\; N_{k,t-1} = \;e({\textbf{N}}_{t-1}) \end{aligned}$$

2 $$\begin{aligned} {L}_{t}&= \; {E}_{t} \; \cdot \; s_{e}({\textbf{N}}_{t-1}) \end{aligned}$$

The juveniles may exist in several age categories (see (3) and (4)) and Fig. 1. The initial juvenile population of age 0 transitions directly from the larvae stage, and its survival may depend on the number of larvae and the adult fish population. The older juvenile populations of age $k=1,...,m-1$ transition from the previous age group into the next, and their survival may be influenced by the number of juveniles within their own age group and the adult population. The rationale behind the survival rate is that (i) juveniles within each age group *k* compete for the same food resource and habitat (ii) the adult population could cannibalize on the lower developmental stanzas, i.e., eggs, larvae and juveniles.

3 $$\begin{aligned} {J}_{0,t}&=\; {L}_{t} \; \cdot \; s_{l}(L_{t}, \; {\textbf{N}}_{t-1}) \end{aligned}$$

4 $$\begin{aligned} {J}_{k,t}&=\; {J}_{k-1,t-1} \; \cdot \; s_{k-1}(J_{k-1,t-1}, \; {\textbf{N}}_{t-1}), \;\;\;\;\;\;\;\;\; k=1, \dots , {m}-1 \end{aligned}$$

Maturation to the youngest age class of adults as described by (5) is either directly from the initial juvenile stage (if $m=0$), or a juvenile group of age $m-1$ (if $m\ge 1$). As described by (6), the adults age linearly. The oldest age class of adults presented in (7) may consist of several age classes, i.e. individuals of any age $\ge n$.

5 $$\begin{aligned} {N}_{{m},t}&=\; \left\{ \begin{array}{ll} {J}_{{m}-1,t-1} \; \cdot \; s_{{m}-1}(J_{{m}-1,t-1}, \; {\textbf{N}}_{t-1})\, , & \;\; {m} \ge 1, \\ {J}_{0,t} \, , & \;\; {m} = 0, \end{array} \right. \end{aligned}$$

6 $$\begin{aligned} N_{k,t}&= \; s_{k-1} \; \cdot \; N_{k-1,{t-1}} , \;\;\;\;\;\;\;\;\; k={m}+1, \dots , {n}-1 \end{aligned}$$

7 $$\begin{aligned} N_{{n},t}&=\; s_{{n}-1} \; \cdot \; N_{{n}-1,t-1} \; + \; s_{n}\; \cdot \; N_{{n},t-1} \end{aligned}$$

All adult age classes $m, \dots , n$ can produce eggs unless fecundity $f_k$ for an age class *k* is equal to 0 (see (1)).

The following conditions guarantee non-negative solutions of (1)–(7) with initial conditions $({\textbf{J}}_0, {\textbf{N}}_0) \in {\mathcal {D}} \subset {\mathbb {R}}_{\ge 0}^{{n}+1}$ (since the product of two non-negative terms is non-negative).

#### Condition 1

For all $k=m, \dots , n$, let $p_k \in (0,1)$ and $f_k\ge 0$ (as in Table 1).

#### Condition 2

For all $i \in \{e,l,0, \dots , {n}-1\}$, let the codomains of functions $s_{i}$ be subsets of (0, 1]. Let the codomain of $s_n$ be a subset of [0, 1].

Furthermore, we assume that $\sum _{k={m}}^{{n}} f_kp_k > 0$ and exclude the case that egg production is equal to zero for all ${\textbf{N}}_t \in {\mathbb {R}}_{\ge 0}^{{n-m}+1}$.

## The SR relationship in multi-stage population dynamics

Given the general DTMM in (1)–(7), we focus in this section on the parent-progeny relationship, as well as mathematical formulations describing this relationship in form of SR functions.

Recruitment $R_t=N_{{m},t}$ is defined as the number of individuals included in stage *m* at time $t \in {\mathbb {N}}_0$, and represented in (5) in the general DTMM. Stock $S_{t}$ is defined as an aggregation of the counts of adults represented by a weighted sum (see (10)).

While the parent-progeny relationship is the general term that describes the relationship between an adult population and their offspring, the traditional SR functions have been defined to mathematically describe the parent-progeny relationship. Whether the existence of SR functions is mathematically valid in a multi-stage framework has not been proven and is the goal of this section.

To establish conditions leading to a closed-form SR function, we proceed in the following way:

**Step 1**: We use the DTMM (in (1)-(7)), representing the full life history cycle, to define the general parent-progeny relationship in Section 3.1.
**Step 2**: We then define and prove conditions under which the general parent progeny relationship can be simplified into a multi-variate SR function (not yet closed-form) (see Section 3.2).
**Step 3**: In Section 3.3, we identify further conditions that allow us to reduce the multi-variate SR function into a closed-from SR function.

In order to establish conditions for steps 2 and 3, we posit two hypotheses for the existence of SR functions, based on our general DTMM.

### Hypothesis 1

$\exists \, \tau \in [1,m+1]$, $\exists \, {\tilde{r}}$ such that for all $t \ge \tau$,

8 $$\begin{aligned} {\tilde{r}}: {\mathbb {R}}_{\ge 0}^{n-m+1} \rightarrow {\mathbb {R}}_{\ge 0} \text { s.t. } {N}_{{m},t} = \; {\tilde{r}}\left( {\textbf{N}}_{t-\tau }\right) . \end{aligned}$$

### Hypothesis 2

$\exists \, \tau \in [1,m+1]$, $\exists \, r$ such that for all $t \ge \tau$,

9 $$\begin{aligned} r: \;&{\mathbb {R}}_{\ge 0} \rightarrow {\mathbb {R}}_{\ge 0} \;\;\; \text { s.t. } \;\;\, {N}_{{m},t} =\; r\left( S_{t-\tau }\right) \, , \end{aligned}$$

where

10 $$\begin{aligned} S_{t}&=\; b({\textbf{N}}_t)=\sum _{k={m}}^{{n}} w_k N_{k,t} \, , \text { with } w_k \ge 0 \, . \end{aligned}$$

The functions ${\tilde{r}}$ and *r* are the multivariate SR, and SR functions, respectively.

### Remark 1

Examples of $S_t$ are the total number of adults (with $w_k=1$ for $k={m}, \dots , {n}$) and the spawning stock biomass (the total weight of fish matured enough to contribute to the reproduction process). In the latter case, $w_k>0$ is the average biomass of an individual in class *k*, respectively.

### Remark 2

The requirement that $\tau \in [1,m+1]$ stems from the observation that $R_t=N_{m,t}$ is in general a function of ${\textbf{N}}_{t-m-1}, \dots , {\textbf{N}}_{t-1}$, as we will see in the following subsection.

If the SR function *r* exists, we may define function ${\tilde{r}} = r \circ b$, the composite function of *r* with the linear function $b: \; {\mathbb {R}}_{\ge 0} \rightarrow {\mathbb {R}}_{\ge 0}$ defined by (10). Function ${\tilde{r}}$ is a multivariate SR function. This allows the following remark.

### Remark 3

Existence of a multivariate SR function is a necessary condition for the existence of a SR function.

### General form of the parent-progeny relationship

The following lemma describes the underlying link between adults and recruitment given by the DTMM.

#### Lemma 1

Let $E_t, L_t, {\textbf{J}}_t, {\textbf{N}}_t$ for $t \ge 0$ satisfy (1)–(7) with $({\textbf{J}}_0, {\textbf{N}}_0) \in {\mathcal {D}}$. Then, $R_t=N_{{m},t}$ is given by (11) for all $t \ge {m}+1$. Here, the probability $j_k$ of survival of eggs to class $k=0, \dots , {m}$ is defined recursively by (12)–(13).

11 $$\begin{aligned}&{N}_{{m},t}= \; e({\textbf{N}}_{t-{m}-1}) \; \cdot \; j_{{m}}({\textbf{N}}_{t-1}, \dots , {\textbf{N}}_{t-{m}-1}) \, \end{aligned}$$

12 $$\begin{aligned}&j_0( {\textbf{N}}_{t-1}) = \; s_{e}({\textbf{N}}_{t-1}) \; \cdot \; s_{l}\Big (e({\textbf{N}}_{t-1}) \cdot s_{e}({\textbf{N}}_{t-1}), \; {\textbf{N}}_{t-1}\Big ) \end{aligned}$$

13 $$\begin{aligned}&j_k( {\textbf{N}}_{t-1} , \dots , {\textbf{N}}_{t-k-1}) = \; j_{k-1}({\textbf{N}}_{t-2}, \dots , {\textbf{N}}_{t-k-1}) \nonumber \\ &\cdot \; s_{k-1} \Big (e({\textbf{N}}_{t-k-1}) \cdot j_{k-1}({\textbf{N}}_{t-2}, \dots , {\textbf{N}}_{t-k-1}),{\textbf{N}}_{t-1} \Big ), \; k = 1, \dots ,{m} \end{aligned}$$

#### Proof

By (1)–(2), we can substitute $e({\textbf{N}}_{t-1})\cdot s_e({\textbf{N}}_{t-1})$ for $L_t$ into (3). We then find $J_{0,t}$ as a function of ${\textbf{N}}_{t-1}$ given by (14). Using (12), we then substitute $j_0({\textbf{N}}_{t-1})$ into (14) to find that (15) holds for $k=0$.

14 $$\begin{aligned} {J}_{0,t}=&\; e({\textbf{N}}_{t-1}) \; \cdot \; s_{e}({\textbf{N}}_{t-1}) \; \cdot \; s_{l}\Big (e({\textbf{N}}_{t-1}) \cdot s_{e}({\textbf{N}}_{t-1}), \; {\textbf{N}}_{t-1}\Big )\end{aligned}$$

15 $$\begin{aligned} {J}_{k,t}=&\; e({\textbf{N}}_{t-k-1}) \; \cdot \; j_k({\textbf{N}}_{t-1}, \dots , {\textbf{N}}_{t-k-1}), \;\; k = 0, \dots ,{m}-1 \end{aligned}$$

We now assume (15) to be true for some integer $k-1\in \{0,\dots , m-2\}$ and find

$$\begin{aligned} {J}_{k,t}=&\; {J}_{k-1,t-1} \; \cdot \; s_{k-1}(J_{k-1,t-1}, \; {\textbf{N}}_{t-1}) \;\;\; \text {(from~}(4)) \\ =&\; e({\textbf{N}}_{t-k-1}) \; \cdot \; j_{k-1}({\textbf{N}}_{t-2}, \dots , {\textbf{N}}_{t-k-1})\; \\&\cdot \; s_{k-1} \Big (e({\textbf{N}}_{t-k-1}) \cdot j_{k-1}({\textbf{N}}_{t-2}, \dots , {\textbf{N}}_{t-k-1}),{\textbf{N}}_{t-1} \Big ) \end{aligned}$$

and thus, by definition (13), we see that (15) also holds for *k*. By mathematical induction, (15) is true for all $k=0, \dots , m-1$.

Similarly to one induction step, we use (5), (13) and (15) with $k=m-1$ to find $N_{{m},t}$ as given by (11). $\square$

We observe that the DTMM describes recruitment $R_t=N_{m,t}$ through (11) as a function of the vectors ${\textbf{N}}_{t-m-1}, \dots , {\textbf{N}}_{t-1}$ of numbers of adults at times $t-m-1, \dots , m-1$. The general parent-progeny relationship involves a series of past states of the adult population at several time steps (from $t-m-1$ to $t-1$).

**Fig. 2:**
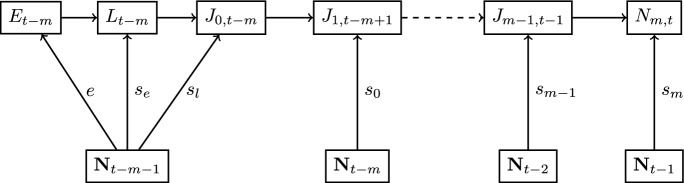
The general parent-progeny relationship for the DTMM. The evolution of individuals spawned at time $t-{m}$ to recruitment at time *t* is given by (1)–(5). Links between stages are represented by arrows. As an example, an arrow from $N_{t-m-1}$ to $E_{t-m}$ represents the assumption that egg production at time step $t-{m}$ is given by function *e* of ${\textbf{N}}_{t-m-1}$ (From Schaarschmidt (2018))

The general form of the parent-progeny relationship admitted by the DTMM is illustrated in Fig. 2. Egg production and the number of larvae and juveniles of age 0 in time step $t-{m}$ are functions of ${\textbf{N}}_{t-m-1}$. Numbers of juveniles of ages $k\ge 1$ are given by functions of the numbers of juveniles in the previous year and age class and the numbers of adults in the previous time step. We obtain recruitment $N_{{m},t}$ at time *t* from the variables ${\textbf{N}}_{t-{m}-1}$, ... , ${\textbf{N}}_{t-1}$. In general, one needs to track the life cycle from the time of spawning to the time of recruitment and use information about each year’s adult population to obtain the SR relationship given by the DTMM.

Using the recursive definition for the probability of survival of eggs to recruitment, we may redefine the multi-stage model in terms of fewer variables.

#### Corollary 1

Let $E_t, L_t, {\textbf{J}}_t, {\textbf{N}}_t$ for $t \ge 0$ be a solution of a DTMM. Then, ${\textbf{N}}_t$ with $t \ge 0$ is a solution of a dynamical system given by (11)–(13) and (6)–(7) with initial condition ${\textbf{N}}_{u} \in \tilde{{\mathcal {D}}}_u \subset {\mathbb {R}}_{\ge 0}^{n-m+1}$ , for suitable $\tilde{{\mathcal {D}}}_u$ and $u=0, \dots , {m}$.

The alternative formulation of the DTMM in Corollary 1 involves only one stage (adults), but the difference equation is of order *m*. We use Corollary 1 to prove the following result.

#### Lemma 2

Let $E_t, L_t, {\textbf{J}}_t, {\textbf{N}}_t$ for $t \ge 0$ be a solution of a DTMM and ${\textbf{N}}_t = {\textbf{N}}$ for all $t \in [0, {m}+1]$. Then, ${\textbf{N}}_t ={\textbf{N}}$ for all $t \ge 0$.

#### Proof

From (11) and ${\textbf{N}}_t = {\textbf{N}}$ for $t =0, \dots ,{m}+1$, it follows that

$$\begin{aligned} N_{{m},{m}+2}\;=\; e({\textbf{N}}_{1}) \; \cdot \; j_{{m}}({\textbf{N}}_{m+1}, \dots , {\textbf{N}}_{1}) \; = \; e({\textbf{N}}_{0}) \; \cdot \; j_{{m}}({\textbf{N}}_{m}, \dots , {\textbf{N}}_{0}) \; = \; N_{{m},{m}+1}. \end{aligned}$$

With (6)–(7) and ${\textbf{N}}_{{m}+1}={\textbf{N}}_{{m}}$, we obtain $N_{k,{m}+2}=N_{k,{m}+1}$, for all $k={m}+1, \dots , n$. Overall, we found that ${\textbf{N}}_t = {\textbf{N}}$ for $t =0, \dots ,{m}+1$ implies ${\textbf{N}}_t = {\textbf{N}}$ for $t =0, \dots ,{m}+2$. Similarly, we observe that if ${\textbf{N}}_t = {\textbf{N}}$ for $t =0, \dots ,{k}+1$ for some $k \ge m$, then ${\textbf{N}}_t = {\textbf{N}}$ for $t =0, \dots ,{k}+2$. By induction, we find ${\textbf{N}}_t = {\textbf{N}}$ for all $t \ge 0$. $\square$

In other words, having a constant adult population for a duration that corresponds to the delay between spawning and recruitment implies the population to be constant for all future times.

### Existence of a multivariate SR function

In this subsection, we investigate, whether the multi-stage model always admits a multi-variate SR function, and state sufficient conditions for the existence of the multi-variate function.

#### Theorem 1

There exist DTMMs without multivariate SR function (as defined in Hypothesis 1).

The proof is by construction, using the following example of a DTMM.

#### Example 1

We consider a dynamical system of form (1)–(7) under assumptions (16)–(19). Let ${\mathcal {D}}$ be a cuboid with positive volume, ${m}=1$ and ${n}=2$. Assumptions (16)–(19) are in accordance with conditions 1–2 which we impose on the DTMM in Sect. 2. The dynamical system is an example of a DTMM.

16 $$\begin{aligned} {E}_{t}&= \; N_{1,t-1}+N_{2,t-1}\end{aligned}$$

17 $$\begin{aligned} s_{e}({\textbf{N}}_{t-1})&=\; s_{l}(L_{t},{\textbf{N}}_{t-1})=s \in (0,1)\end{aligned}$$

18 $$\begin{aligned} s_{0}(J_{k,t-1}, \; {\textbf{N}}_{t-1})&=\; e ^{-2N_{1,t-1}}\end{aligned}$$

19 $$\begin{aligned} s_1&= 0.2 \; \text { and } s_2 = 0 \end{aligned}$$

Substituting assumptions (16)–(19) into the DTMM, the dynamics for juveniles and adults are given by (20)–(22) with (21) for $R_t=N_{1,t}$.

20 $$\begin{aligned} J_{0,t}&= \; (N_{1,t-1}+N_{2,t-1}) s^2 \end{aligned}$$

21 $$\begin{aligned} N_{1,t}&=\; J_{0,t-1} e ^{-2N_{1,t-1}} \end{aligned}$$

22 $$\begin{aligned} N_{2,t}&=\; 0.2 N_{1,t-1} \end{aligned}$$

#### Proof of Theorem 1

Our goal is to show that there exist solutions to the dynamical system that are such that a multivariate SR function cannot exist for any $\tau \in [1,m+1]=[1,2]$.

Let $({\textbf{J}}^1_0, {\textbf{N}}^1_0)$, $({\textbf{J}}^2_0, {\textbf{N}}^2_0) \in {\mathcal {D}}$ such that ${\textbf{N}}_0^1 = {\textbf{N}}_0^2$ and $J_{0,0}^1 \ne J_{0,0}^2$. These vectors exist in ${\mathcal {D}}$ since the cuboid is assumed to have positive volume. Denote by $({\textbf{J}}^1_t, {\textbf{N}}^1_t)$ and $({\textbf{J}}^2_t, {\textbf{N}}^2_t)$ the solutions to (20)–(22) with initial condition $({\textbf{J}}^1_0, {\textbf{N}}^1_0)$ and $({\textbf{J}}^2_0, {\textbf{N}}^2_0)$, respectively.

**The case **$\varvec{\tau = 1}$. For this case, we let $t=1$ and consider recruitment $N_{1,1}$ as given by (21). With the assumptions $N_{1,0}^1= N_{1,0}^2$ and $J_{0,0}^1 \ne J_{0,0}^2$ about the initial conditions, we see that $N_{1,1}^1 \ne N_{1,1}^2$. As ${\textbf{N}}_0^1 = {\textbf{N}}_0^2$ but $N_{1,1}^1 \ne N_{1,1}^2$, a multivariate SR function ${\tilde{r}}: {\mathbb {R}}_{\ge 0}^{n-m+1} \rightarrow {\mathbb {R}}_{\ge 0}$ s.t. ${N}_{1,t} ={\tilde{r}}\left( {\textbf{N}}_{t-1}\right)$ for all $t \ge 1$ cannot exist.

**The case **$\varvec{\tau = 2}$. Now, let $t=2$. We substitute $J_{0,0}$ as defined by (20) into (21) for $N_{1,t}$ and obtain (23) for recruitment at time $t=2$. Since ${\textbf{N}}_0^1 = {\textbf{N}}_0^2$ and $N_{1,1}^1 \ne N_{1,1}^2$, we observe that $N_{1,2}^1 \ne N_{1,2}^2$. Thus, a function ${\tilde{r}}: {\mathbb {R}}_{\ge 0}^{n-m+1} \rightarrow {\mathbb {R}}_{\ge 0}$ s.t. ${N}_{1,t} ={\tilde{r}}\left( {\textbf{N}}_{t-2}\right)$ for all $t \ge 2$ cannot exist.

23 $$\begin{aligned} N_{1,2} \; = \; (N_{1,0}+N_{2,0}) s^2 e ^{-2N_{1,1}} \end{aligned}$$

Summarizing, we have now shown that for $\tau = 1$ and $\tau = 2$, the DTMM (20)–(22) has solutions, for which there cannot exist any function ${\tilde{r}}: {\mathbb {R}}_{\ge 0}^n \rightarrow {\mathbb {R}}_{\ge 0}$ s.t. ${N}_{1,t} ={\tilde{r}}\left( {\textbf{N}}_{t-\tau }\right)$ for all $t \ge \tau$. Hypothesis 1 does not hold for Example 1.

$$\square$$

In the following, we consider a set of properties of Example 1. We consider them as logical statements and use terminology described e.g. in Rosen (2012).

#### Propositions

Define the following set of propositions, which are said to be true if they are true for all solutions $E_t, L_t, {\textbf{J}}_t, {\textbf{N}}_t$, $t \ge 0$ of a DTMM.

1. $${\textbf{N}}_{t}= {\textbf{N}}_0$$ for all $$t \in [0,{m}+1]$$,
2. $s_{k}(J_{k,t}, \; {\textbf{N}}_t) = s_{k}(J_{k,t})$ for all $k=0, \dots {m}-1$ and for all $t \ge 0$,
3. ${m}=0$, i.e. $N_{{m},t}=J_{0,t}$ is given by (3).

A biological interpretation of (C3) is that there is only one cohort of juveniles. Then, recruitment of fish spawned at time *t* occurs at time $t+1$.

##### Remark 4

For Example 1, the logical solution of ((C1) $\vee$ (C2) $\vee$ (C3)) is false.

##### Proof

The verification that (C1) evaluates as false follows directly from the proof of Theorem 1. Since there exists ${\tilde{t}} \ge 0$ such that $N_{1,{\tilde{t}}} \ne N_{1,{\tilde{t}}+1}$ and (18) holds, (C2) cannot be true. The negation of (C3) holds by definition of the dynamical system. $\square$

In other words, the multi-stage model from Example 1 without multivariate SR function has none of the properties (C1), (C2) or (C3).

#### Sufficient conditions for the existence of a multivariate SR function

##### Theorem 2

For all DTMMs, each of the conditions (C1), (C2) and (C3) is individually sufficient for the existence of a multivariate SR function (Hypothesis 1). Function ${\tilde{r}}$ is given by (24) and $\tau ={m}+1$.

24 $$\begin{aligned} {N}_{{m},t} =&\; e({\textbf{N}}_{t-{m}-1}) \; \cdot \; j_{{m}}({\textbf{N}}_{t-{m}-1})\, , \; \text {for all } t \ge m+1\, . \end{aligned}$$

For the proof, we use Lemma 1 and (11)–(13) given therein. For the convenience of the reader, the equations are re-stated here,

11' $$\begin{aligned}&{N}_{{m},t}= \; e({\textbf{N}}_{t-{m}-1}) \; \cdot \; j_{{m}}({\textbf{N}}_{t-1}, \dots , {\textbf{N}}_{t-{m}-1}) \, \end{aligned}$$

12' $$\begin{aligned}&j_0( {\textbf{N}}_{t-1}) = \; s_{e}({\textbf{N}}_{t-1}) \; \cdot \; s_{l}\Big (e({\textbf{N}}_{t-1}) \cdot s_{e}({\textbf{N}}_{t-1}), \; {\textbf{N}}_{t-1}\Big ) \end{aligned}$$

13' $$\begin{aligned}j_k(& {\textbf{N}}_{t-1} , \dots , {\textbf{N}}_{t-k-1}) = \; j_{k-1}({\textbf{N}}_{t-2}, \dots , {\textbf{N}}_{t-k-1}) \\ \cdot & \; s_{k-1} \Big (e({\textbf{N}}_{t-k-1}) \cdot j_{k-1}({\textbf{N}}_{t-2}, \dots , {\textbf{N}}_{t-k-1}),{\textbf{N}}_{t-1} \Big ), \; k = 1, \dots ,{m} \end{aligned}$$

##### Proof

Assume that (C1) holds. With Lemma 2, we obtain that ${\textbf{N}}_t={\textbf{N}}$ for all $t \ge 0$. By (11) for recruitment in Lemma 1, we can conclude that $N_{{m},t}$ is given by (24), for all $t \ge {m}+1$.

Using the recursive definition (12)-(13) for probability $j_k({\textbf{N}}_t)$, we observe that (C2) implies that $j_k$ is given by (25) for $k=1, \dots ,{m}$ and $t \ge k+1$. In this case and due to (11), we obtain (24), for all $t \ge {m}+1$.

25 $$\begin{aligned} j_k({\textbf{N}}_{t-k-1}) = \; j_{k-1}({\textbf{N}}_{t-k-1}) \; \cdot \; s_{k-1} \Big (e({\textbf{N}}_{t-k-1}) \cdot j_{k-1}( {\textbf{N}}_{t-k-1})\Big ) \end{aligned}$$

Proposition (C3) and $m=0$ implies the existence of a multivariate SR function of form (24) for all $t \ge {m}+1$ by (11). $\square$

**Fig. 3:**
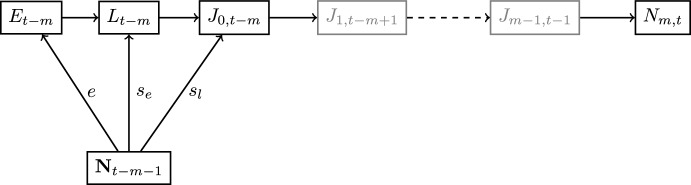
The multi-variate SR function for the DTMM. Here, the evolution of individuals spawned at time $t-{m}$ to recruitment at time *t* is given by equations (1)–(5) under condition (C2)$\vee$(C3). The resulting parent-progeny relationship is a multi-variate SR function (as recruitment is a function of ${\textbf{N}}_{t-m-1}$). The gray color of some nodes represents the fact that under (C3), i.e., $m=0$, the nodes $J_{1,t-m+1}$ and $J_{m-1,t-1}$ are not included in the graph (adapted from Schaarschmidt (2018))

The simplification of the general parent-progeny relationship from Fig. 2 into a multi-variate SR function is illustrated in Fig. 3. The biological interpretation of Theorem 2 is that we can be sure to have a multi-variate SR function, if either

- the system is at a stable fixed point,
- survival rates of juveniles are constant with respect to the numbers of adults, or
- there is no more than one time step delay between spawning and recruitment.

### Existence of a SR function

We now state sufficient conditions for a multi-variate SR function to have the characteristic form of a SR function and investigate what happens, if any of the conditions are violated.

For the DTMM, we define the following propositions. Here, we use linear function $b: \; {\mathbb {R}}^{n-m+1}_{\ge 0} \rightarrow {\mathbb {R}}_{\ge 0}$ defined by (10).

- $$\exists$$ $${\tilde{e}}: {\mathbb {R}}_{\ge 0} \rightarrow {\mathbb {R}}_{\ge 0}$$ s.t. $$e({\textbf{x}})={\tilde{e}}(b({\textbf{x}}))$$, for all $${\textbf{x}} \in {\mathbb {R}}_{\ge 0}^{n-m+1}$$
  . A biological interpretation of (C4)(a) is that egg production can be adequately represented by a function of the weighted sum
  $S_t$ of numbers of adults.
- $$\exists$$ $${\tilde{s}}_e: {\mathbb {R}}_{\ge 0} \rightarrow (0,1]$$ s.t. $$s_e({\textbf{x}})={\tilde{s}}_e(b({\textbf{x}}))$$, for all $${\textbf{x}} \in {\mathbb {R}}_{\ge 0}^{n-m+1}$$
  . In other words, survival of eggs can be described by a function of
  $$S_t$$.
- $$\exists$$ $${\tilde{s}}_l: {\mathbb {R}}_{\ge 0}^2 \rightarrow (0,1]$$ s.t. $$s_l(y,{\textbf{x}})={\tilde{s}}_l(y,b({\textbf{x}}))$$ for all $${\textbf{x}} \in {\mathbb {R}}_{\ge 0}^{n-m+1}$$, $$y \in {\mathbb {R}}_{\ge 0}$$.

This translates to mean that survival of larvae can be represented by a function of the stock $S_t$.The codomains of ${\tilde{s}}_e$ and ${\tilde{s}}_l$ are chosen in line with assumption 2 on the DTMM.

#### Remark 5

Let $E_t, L_t, {\textbf{J}}_t, {\textbf{N}}_t$, $t \ge 0$, be a solution of a DTMM and assume that (C1) is true. From Lemma 2, we know that (C1) implies $\mathbf {{N}}_{t}={\textbf{N}}_{0}$ for all $t \ge 0$. Thus, ${N}_{m,t}={N}_{m,0}$ and $S_t=S_0$ for all $t \ge 0$ and it is possible to construct a SR function $r: \; {\mathbb {R}}_{\ge 0} \rightarrow {\mathbb {R}}_{\ge 0}$ such that ${N}_{{m},t} =\; r\left( S_{t-{(m+1)}}\right)$ for all $t \ge m+1$, i.e., Hypothesis 2 is true.

#### Theorem 3

Consider a DTMM, for which the logic solution of (C3) is true. Then, violation of exactly one of the logical statements (C4)(a)–(c) implies that no SR function exists.

#### Proof

Consider a DTMM such that (C4)(b) and (C4)(c) hold and (C4)(a) is violated. Assume $m=0$, i.e. a multivariate SR function exists. From the proof to Lemma 1, we know that recruitment $N_{{m},t}$ is given by (14), for $t \ge 1$. With (C4)(b) and (C4)(c), assuming the existence of a SR function would imply (26)–(27) for all $t \ge 1$. This would be a contraction to (C4)(a) being false. Thus, this DTMM cannot have any SR function.

26 $$\begin{aligned} {N}_{0,t} =&\; e({\textbf{N}}_{t-1}) \; \cdot \; {\tilde{s}}_{e}(b({\textbf{N}}_{t-1})) \; \cdot \; {\tilde{s}}_{l}(b({\textbf{N}}_{t-1}))= \;r(b({\textbf{N}}_{t-1})) \end{aligned}$$

27 $$\begin{aligned} e({\textbf{N}}_{t}) =&\; \left\{ \begin{array}{ll} {\tilde{s}}_{e}(b({\textbf{N}}_{t})) \; \cdot \; {\tilde{s}}_{l}\left( b({\textbf{N}}_{t})\right) \;/ \; r(b({\textbf{N}}_{t})), & \; r(b({\textbf{N}}_{t})) >0 \\ 0 , & \text { else } \end{array} \right. \end{aligned}$$

Similarly, we can define DTMMs, for which either (C4)(b) or (C4)(c) is violated, but (C4)(a) holds and there exists no SR function. $\square$

We have now proved that a multi-variate SR function is not required to have the characteristic form of a SR function unless we impose structural conditions on the functions representing egg production and survival rates of eggs and larvae. However, we will now see that the combination of these three conditions ensures the existence of a SR function.

#### Sufficient conditions for the existence of a SR function

##### Theorem 4

For all DTMMs, the logic solution of ((C4) $\wedge$ ((C2) $\vee$ (C3))) $\vee$ (C1) is sufficient for the existence of a SR function *r* given by (28) with $\tau = {m}+1$. Here, we use ${\tilde{j}}_k:{\mathbb {R}}_{\ge 0} \rightarrow {\mathbb {R}}_{\ge 0}$ s.t. ${j}_k = {\tilde{j}}_k \circ b$ for $k=0, \dots , m$.

28 $$\begin{aligned} {N}_{{m},t}&= \; r(S_{t-m-1}) = \; {\tilde{e}}(b({\textbf{N}}_{t-{m}-1})) \; \cdot \; {\tilde{j}}_{{m}}(b({\textbf{N}}_{t-k-1})) \; \text { for all } t \ge m+1 \, . \end{aligned}$$

##### Proof

Let (C4) $\wedge$ ((C2) $\vee$ (C3)) be true and $E_t, L_t, {\textbf{J}}_t, {\textbf{N}}_t$, $t \ge 0$, a solution of a DTMM. Then, by Theorem 2 and Lemma 1, we know that $N_{m,t}$ is given by (12) and (24)–(25), for $t \ge m + 1$.

Due to (C4), we get (29) and may represent probability $j_0$ as a function of $S_{t-m-1}=b({\textbf{N}}_{t-m-1})$. Using (C4), we recursively find that (30) holds for all $k=1, \dots , {m}$ and ${\textbf{x}} \in {\mathbb {R}}^{{n}-{m}+1}$. From Theorem 2, we see that the SR function is in this case defined by (28).

29 $$\begin{aligned} j_0&({\textbf{x}}) = \; {\tilde{s}}_{e}(b({\textbf{x}})) \; \cdot \; {\tilde{s}}_{l}(b({\textbf{x}})) = \; {\tilde{j}}_0(b({\textbf{x}})) \end{aligned}$$

30 $$\begin{aligned} j_k&( {\textbf{x}}) =\; {\tilde{j}}_{k-1}(b({\textbf{x}})) \; \cdot \; s_{k-1} \Big ({\tilde{e}}(b({\textbf{x}})) \cdot {\tilde{j}}_{k-1}(b({\textbf{x}}))\Big ) =\; {\tilde{j}}_k( b({\textbf{x}})) \end{aligned}$$

The existence of a SR function, if (C1) evaluates as true, follows directly from Remark 5. $\square$

**Fig. 4:**
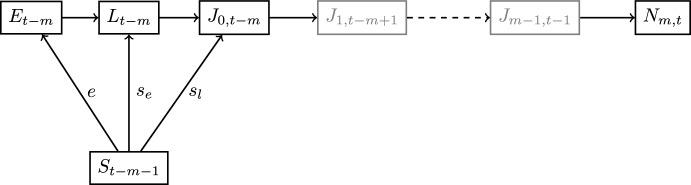
The closed-form SR function for the DTMM. The evolution of individuals spawned at time $t-{m}$ to recruitment at time *t* given is by equations (1)–(5) assuming (C4) $\wedge$ ((C2) $\vee$ (C3)) to be true. The resulting parent-progeny relationship is a closed-form SR function (since $N_{m,t}$ is a function of $S_{t-m-1}$). The gray color of some nodes represents the fact that under (C3), i.e., $m=0$, the nodes $J_{1,t-m+1}$ and $J_{m-1,t-1}$ vanish (adapted from Schaarschmidt (2018))

The simplification of the general parent-progeny relationship for the DTMM into a closed-form SR function is illustrated in Fig. 4.

## Discussion

Age- and stage-structure of fish populations may vary with time. Hence, there is a need to consider all stanzas in the life history to realistically define the transition from spawning to recruitment. Consistent with this consideration, we have presented a DTMM describing a generic life cycle of fish with four stages and several age classes. Based on this model, we have demonstrated two important principles concerning a functional presentation of the parent-progeny relationship for fish. Firstly, we have proven that in general, both the multi-variate and closed-form SR functions are limiting, because they do not track the age- and stage-structure from spawning to recruitment. Secondly, we defined necessary and sufficient conditions for the existence of a closed-form SR function. In other words, we outlined assumptions that enable the biological processes driving recruitment to be represented through a SR function, i.e., a mathematical function involving one or more variables.

The closest modeling approach to that presented in this paper is by Touzeau and Gouzé (1998) and Schaarschmidt et al (2018). Our work presents an extension of these previous approaches in considering all life history stages, instead of a broad classification of the population into prerecruits (eggs, larvae, and juveniles) and adults. We have represented both the fast processes (spawning, egg and larval survival) and the slower processes (ageing of juveniles) which occur prior to recruitment.

Assuming that conditions (C2) and (C4) are true, the existence of a SR function is guaranteed by Theorem 4. These two conditions are implied when one assumes that (i) the number of individuals in the first life stage is a function of *S*, and (ii) the number of individuals in all subsequent stages is a function of the number of individuals in the previous stage. The approach by Paulik (1973) and Brooks and Powers (2007) in investigating the functional forms of parent-progeny relationships admitted by multi-stage models, was underpinned by these assumptions. It is known that cannibalism can be a limiting factor for survival (Bogstad et al 2016). Inclusion of cannibalism and age-structure in our model, extends the approaches by Paulik (1973) and Brooks and Powers (2007).

We found the existence of a multi-variate SR function to be premised on either the population being at a stable fixed point, recruitment being defined as the transition rate to age class 0, or mortality of juveniles being constant with respect to the number of adults. These conditions are considered sufficient.

We proved that the existence of a multi-variate SR function is necessary, but not sufficient, for the existence of a SR function of a single variable. To ensure that the parent-progeny relationship has the conventional form of a SR function, additional conditions must be imposed on the structure of the functions representing egg production and survival of eggs and larvae. It is required that the three processes are given by functions of the same weighted sum of numbers of adults (e.g., the total number of adults or the spawning stock biomass).

When defining recruitment as the transition rate to age class 0, the evolution of all prerecruit (eggs, larvae, juveniles) happens at faster rates than aging and mortality of adult fish. This is in line with the observation made by Schaarschmidt et al (2018), who found fast prerecruit dynamics to be a prerequisite for the existence of a multi-variate SR function.

Previously, SR functions have been derived assuming age-independence of parameters related to egg production and survival of prerecruits (Quinn and Deriso 1999; Touzeau and Gouzé 1998). In this case, all age classes in the adult population contribute homogeneously to egg production and survival and the sufficient condition (C4) for the existence of an SR function holds. However, assuming age-independence of parameters is but one way of ensuring that (C4) holds.

In summary, we observe that by choosing a generic modeling framework, we found necessary and sufficient conditions for the existence and structure of the SR function with broad applicability. In this sense, our results extend and unify previous observations made under a variety of modeling assumptions.

Our DTMM approach offers valuable insights into recruitment dynamics but includes simplifications that may affect real-world applicability. Biological processes like spawning, maturation, and survival are continuous by nature, but we discretized them for mathematical analysis and to derive SR function conditions. This may overlook temporal and spatial variations in these processes. Future research could integrate continuous-time dynamics, potentially using differential equations. Incorporating spatially explicit data and time-varying parameters would also better capture survival and maturation variability influenced by habitat, predation, and competition. Hybrid models combining discrete and continuous elements, as well as further stochastic details, could enhance the realism and predictive power of the DTMM. A further step towards capturing the complexities of recruitment processes would be to account for environmental factors that influence fish populations. Recruitment models that include environmental covariates have been described in the fisheries literature (as reviewed by Subbey et al (2014)). While simplified, our approach provides a robust framework for understanding the relationship between adult fish populations and recruitment.

We have considered an age-structured population though the framework can apply to populations structured by length or weight. The existence of a multi-variate SR function remains valid regardless of whether the adult population is structured according to age, length or weight. However, a univariate SR function in a closed-form is dependent on additional conditions.

From a modeling perspective, the multi-stage approach enhances the realism of simulations by accounting for all life stages and their inter-dependencies. It allows for a more comprehensive assessment of how various pressures (e.g., fishing intensity and environmental changes) affect each stage and, consequently, overall recruitment. This approach can potentially help identify biological factors that influence early life stages, which are critical for predicting future stock sizes and assessing potential risks to recruitment.

By delineating the necessary and sufficient conditions for a valid SR function, the multi-stage modeling approach could help clarify the contexts in which traditional SR models may fail, thereby reducing uncertainty in stock assessments. Alternatively, starting with a multi-stage model, one could determine when a SR function may be a valid simplification, thus reducing model complexity in stock assessment. This way, we also address challenges related to data collection: if conditions for the existence of a multi-variate SR function prevail, any challenges related to prerecruit data may safely be omitted.

For fisheries management, the insights from this paper highlight the potential risks of relying solely on traditional SR functions, which may lead to over- or underestimations of recruitment under specific conditions. The proposed multi-stage model offers a more nuanced framework, potentially enabling managers to pinpoint critical bottlenecks in the life cycle that could be addressed to enhance recruitment. This approach supports more targeted interventions, such as setting quotas or establishing protected areas tailored to specific life stages. Given the complexity of life cycles in many fish species, this model provides an approach to developing management strategies that align more closely with species-specific dynamics.

## Data Availability

This is not applicable to this article as no datasets were generated or analyzed during the current study.
